# Factores psicosociales y rendimiento laboral en colaboradores de una institución educativa privada, Lima, 2021*

**DOI:** 10.15649/cuidarte.2738

**Published:** 2023-12-25

**Authors:** María Magdalena Díaz Orihuela, Janett Virginia Chávez Sosa, Luz Victoria Castillo Zamorra, Dajana Lyz Marquez Arcce, Jhon David Tantajulca Zuta, David Bryan Santamaría Gutiérrez

**Affiliations:** 1 . Enfermera docente de la Universidad Peruana Unión, E.P. Enfermería, Lima, Perú. Email: magi@upeu .edu.pe Universidad Peruana Unión Universidad Peruana Unión Lima Peru magi@upeu .edu.pe; 2 . Enfermera docente de la Universidad Peruana Unión. E.P. Enfermería, Lima, Perú. Email: viki16@upeu .edu.pe Universidad Peruana Unión Universidad Peruana Unión Lima Peru viki16@upeu .edu.pe; 3 . Enfermera docente de la Universidad Peruana Unión. E.P. Enfermería, Lima, Perú Email: viky@upeu.edu.pe Universidad Peruana Unión Universidad Peruana Unión Lima Peru viky@upeu.edu.pe; 4 . Enfermera administrativa en Clínica Virgen de las Mercedes. Perú Lima. Email: dajanamarquez@upeu.edu.pe Clínica Virgen de las Mercedes Perú dajanamarquez@upeu.edu.pe; 5 . Enfermero asistencial del Puesto de Salud I-2 Ninahuaya, Loreto, Perú. Email: ihontantajulcazuta@gmail.com Puesto de Salud I-2 Ninahuaya Loreto Perú ihontantajulcazuta@gmail.com; 6 . Enfermero administrativo en Clínica Good Hope. Perú Lima. Email: davidsantamaria@upeu.edu.pe Clínica Good Hope Lima Perú davidsantamaria@upeu.edu.pe

**Keywords:** Salud Laboral, Rendimiento Laboral, Enfermedades Ocupacionales, Satisfacción en el Trabajo, Occupational Health, Work Performance, Psychosocial Aspects, Occupational Diseases, Job Satisfaction, Médicos do Trabalho, Desempenho Profissional, Aspectos psicossociais, Doengas Profissionais, Satisfagáo no Emprego

## Abstract

**Introducción:**

La actividad laboral de las personas siempre ha transformado al mundo, sin embargo, ha generado muchos riesgos y enfermedades. Asimismo, el trabajo ha sido afectado por la globalización, la tecnología y la crisis sanitaria por la COVID-19. Generando, transformaciones en las estructuras del empleo y en las condiciones del trabajo, tornándose más exigente en el área laboral, estas condiciones han predispuesto factores psicosociales negativos que amenaza la salud; ocasionando enfermedades y accidentes laborales, y como consecuencia un deficiente rendimiento laboral.

**Objetivo:**

Determinar la relación entre los factores psicosociales y el rendimiento laboral en colaboradores de una institución educativa privada de Lima.

**Metodología:**

Enfoque cuantitativo, de diseño no experimental, corte transversal y de tipo correlacional. En el estudio participaron 120 colaboradores de la institución educativa privada, según el muestreo no probabilístico; como instrumento se aplicó el Cuestionario de “Factores Psicosociales en el Trabajo” y el “El cuestionario de Rendimiento Laboral Individual” compartido digitalmente través del correo electrónico.

**Resultados:**

Del total de los encuestados, el 52,50% manifestaron tener una buena percepción de los factores psicosociales; sin embargo, se obtuvo una mala percepción en las dimensiones exigencia laboral con el 63,30% y satisfacción con la remuneración del rendimiento con el 52,50%; con respecto a la variable rendimiento laboral, el 75,00% demostró un nivel alto.

**Conclusión:**

Los trabajadores que indicaron una alta exigencia laboral tuvieron 11 veces mayor probabilidad de presentar un alto rendimiento en su trabajo (OR:11,03; IC 95%: 1,71-17,95).

## Abstract (en)

**Introduction:**

People's work has always transformed the world; however, it has also posed many risks and diseases. Likewise, work has been affected by globalization, technology, and the COVID-19 health crisis. There have been transformations in employment structures and working conditions, which have become more demanding. These conditions have predisposed negative psychosocial factors that threaten health, causing occupational illnesses and accidents and, consequently, poor work performance.

**Objective:**

To determine the relationship between psychosocial factors and work performance in employees of a private educational institution in Lima.

**Methods:**

Quantitative approach, non-experimental, cross-sectional, and correlational design. A total of 120 employees of the private educational institution participated in the study, according to a non-probabilistic sampling method. The Psychosocial Factors at Work Questionnaire and the “Individual Work Performance Questionnaire,” shared digitally via e-mail, were used.

**Results:**

Of the total respondents, 52.50% had a good perception of psychosocial factors; however, a poor perception was identified in the dimensions “work demands” (63.30%) and “satisfaction with performance compensation” (52.50%).

**Conclusion:**

Workers who indicated high work demands were 11 times more likely to have high work performance (OR: 11.03; 95% CI: 1.71-17.95).

## Resumo

**Introdugáo:**

A atividade de trabalho das pessoas sempre transformou o mundo, no entanto, gerou muitos riscos e doengas. Da mesma forma, o trabalho tem sido afetado pela globalizagáo, pela tecnologia e pela crise sanitária da COVID-19. Estas condigóes predispuseram fatores psicossociais negativos que ameagam a saúde, causando doengas e acidentes no trabalho e, como consequéncia, um mau desempenho no trabalho.

**Objetivo:**

Determinar a relagáo entre fatores psicossociais e o desempenho no trabalho dos funcionários de uma instituigáo de ensino privada em Lima.

**Metodologia:**

Abordagem quantitativa, projeto náo-experimental, transversal e correlacional. O estudo envolveu 120 funcionários da instituigáo privada de ensino, de acordo com amostragem náo-probabilística; o questionário "Fatores Psicossociais no Trabalho" e o "Questionário de Desempenho Individual do Trabalho", compartilhado digitalmente por e-mail, foram utilizados como instrumentos.

**Resultados:**

Do número total de entrevistados, 52,50% afirmaram que tinham uma boa percepgáo dos fatores psicossociais; no entanto, foi obtida uma má percepgáo nas dimensóes das demandas do trabalho com 63,30% e satisfagáo com a remuneragáo por desempenho com 52,50%; com relagáo ao desempenho variável do trabalho, 75,00% mostraram um alto nivel.

**Conclusao:**

Os trabalhadores que indicaram altas exigencias de trabalho tinham 11 vezes mais probabilidade de ter alto desempenho no trabalho (OR: 11,03; 95% CI: 1,71-17,95).

## Introducción

La actividad laboral de las personas siempre ha transformado al mundo, sin embargo, ha generado muchos riesgos y enfermedades correspondientes al trabajo. Asimismo, a lo largo de los años el universo laboral ha sido afectado por la globalización, el desarrollo de tecnologías y, por último, recientemente esta situación ha sido incrementada por la crisis sanitaria originado por el virus de la COVID-19. En este contexto, se produjeron diversos cambios en las empresas o instituciones tanto públicas como privadas, así como transformaciones en las estructuras del empleo, selección del personal y las condiciones laborales, tornándose aún más exigente en el área laboral, estas condiciones laborales han predispuesto factores psicosociales considerándose una amenaza para la salud, ocasionando enfermedades y accidentes laborales^1^.

Cada año aproximadamente 2,78 millones de trabajadores fallecen por accidentes y enfermedades relacionadas al trabajo, siendo el 86,3% enfermedades de origen laboral y el 13,7% accidentes mortales; haciendo un total de 7,500 muertes por día^2^. Por otra parte, la Organización Internacional del Trabajo (OIT), menciona que las enfermedades y los accidentes ocupacionales generan un costo directo o indirecto del 4% al PBI (producto bruto interno); siendo 2,8 billones de dólares aproximadamente, cifra que podría elevarse en un 6% en ciertos países^3^. De igual manera, el Ministerio del Trabajo y Promoción del Empleo reportó que, en el Perú, del total de notificaciones al primer trimestre del año 2022, “el 97,43% corresponde a accidentes de trabajo no mortales, el 0,43% a accidentes mortales, el 1,99% a accidentes peligrosos y 0,15% a enfermedades ocupacionales”^4^. Como causa de las deficiencias en la seguridad y salud en el trabajo de las empresas.

Las nuevas formas de trabajo han adquirido nuevos riesgos y enfermedades laborales. Estas “nuevas formas de organización del trabajo implican riesgos y oportunidades de diferente naturaleza”. Es por ello, que varios trabajadores se ven sujetos a diversas exigencias las cuales en muchas ocasiones perjudican su salud, vida personal y laboral de manera inapropiada y deficiente^5^. Un estudio realizado en 35 países por la Agencia Europea para la Seguridad y la Salud en el Trabajo - AEST (2017), resulto que un 43% de los encuestados reportaron tener problemas de salud y un 15% de ellos presentan ansiedad; así casi todos los problemas de salud se relacionan directamente con los factores psicosociales negativos como el entorno físico y social y, a la intensidad de trabajo^6^. Del mismo modo, un estudio realizado en Argentina, por la Superintendencia de Seguridad Social, menciona que el 44% de empleados tienen tendencia a factores psicosociales de riesgo en el trabajo^7^.

Por consiguiente, es muy importante considerar la salud ocupacional del trabajador, siendo indispensable la garantía de la seguridad y salud al momento de tratar el impacto de la pandemia por COVID-19. Sin embargo, la modalidad y condiciones de trabajo cambiaron de manera considerable, impulsando a nuevos desafíos psicosociales para la mejora y bienestar de los trabajadores^8^. Asimismo, según el Instituto Nacional de Estadística e Informática (INEI), refiere que en el Perú el empleo formal representa un 27,4% del empleo total, mientras que el empleo informal representa el 72,6% de los trabajadores, considerándose de mayor riesgo laboral, falta de seguridad y exposición a personas vulnerables^9^. En consecuencia, las nuevas formas de trabajo han adquirido nuevos riesgos y enfermedades laborales. Estas “nuevas formas de organización del trabajo implican riesgos y oportunidades de diferente naturaleza” las cuales en varias ocasiones dañan su vida privada, su salud y su vida laboral de manera inapropiada y deficiente^10^.

La Organización Internacional del Trabajo (OIT) refiere que “un trabajo adecuado y justo, brinda bienestar y salud, pero un trabajo sobrecargado y con un ambiente negativo, puede llegar a perjudicar a la persona dañando su salud, incrementando la posibilidad de que ocurran accidentes dentro o fuera de la organización afectando el rendimiento y el ambiente de trabajo en general”^11^. Asimismo, la teoría propuesta por Nola Pender refiere que, para tener bienestar es necesario comprender el comportamiento del individuo y su salud, al mismo tiempo orientadas a adoptar conductas saludables^12^.

El presente artículo proporciona la pauta para que su aplicación se replique en otras instituciones similares, dando a conocer que tipos de riesgos psicosociales existen en una institución educativa privada y en sus trabajadores, para tomar las medidas necesarias. Así el interés de efectuar el estudio, es relacionar los factores psicosociales que están presentes y en qué manera afecta en el rendimiento laboral de los colaboradores, para así brindar una visión integral del personal y a la vez mantener a la vanguardia aquello que afecta la salud laboral. Del mismo modo, los factores psicosociales provocados por el COVID-19, estrés laboral, accidentes laborales, entorno laboral, etc., afectan en el rendimiento de los colaboradores en el desempeño de sus labores la práctica que ello realizan; en ese contexto se plantea la siguiente formulación del problema ¿Existe relación entre los factores psicosociales y rendimiento laboral en colaboradores de una institución educativa privada, Lima, 2021?

## Materiales y Métodos

La información validada se exportó al paquete estadístico R de acceso libre, para el almacenamiento de los datos fue en el formato CSV la cual se encuentra almacenada en el repositorio de la una universidad privada el acceso En el repositorio de tesis, de la universidad Peruana Unión.^13^

### Tipo de estudio

La investigación es de enfoque cuantitativo, diseño no experimental, de corte transversal, ya que no hubo manipulación de las variables; tipo y nivel correlacional, porque se describió la relación entre las variables^13^.

### Población y muestra

Se recolecto la información requerida de 120 colaboradores de una institución educativa privada de Lima. La muestra se efectuó mediante el muestreo no probabilístico, por conveniencia de tipo censal. Se incluyeron a los trabajadores de ambos sexos, de nacionalidad Peruana y que llevaran laborando en la institución por lo menos 3 meses, asimismo, se excluyó al personal que se encontrara de vacaciones o licencia, de nacionalidad extranjera y que no aceptaran participar del estudio.

### Instrumentos de recolección de datos

Se utilizaron dos cuestionarios denominados “Cuestionario de Factores psicosociales en el trabajo” para la variable “A” y el “cuestionario de rendimiento laboral individual” para la variable “B” para recolección de datos. Como técnica utilizada los mismos que se realizaron mediante la encuesta online para ambas variables.

### Cuestionario de Factores psicosociales en el trabajo

Para la obtención de datos para la variable “A” se utilizó el Cuestionario de “Factores Psicosociales en el Trabajo”, elaborado por Noemí Silva Gutiérrez en el 2004 en México y validado en Perú por Pando, Varilla, Aranda y Elizalde en el 2016, con una confiabilidad del Alpha de Cronbach de 0,900 lo que indica una fiabilidad optima^14^. El instrumento cuenta con 46 ítems con formato de respuesta tipo Likert que va desde Nunca (0), Casi Nunca (1), Algunas Veces (2), Casi Siempre (3) y Siempre (4). Dividido en 7 dimensiones: Condición del lugar de trabajo (con 9 ítems), Carga de trabajo (5 ítems), Contenido y características de la tarea (7 ítems), Exigencias laborales (7 ítems), Papel laboral y desarrollo de la carrera (6 ítems), Interacción social y aspectos organizacionales (9 ítems) y Satisfacción con la Remuneración del Rendimiento (3 ítems).

### Cuestionario de Rendimiento Laboral Individual

Para la variable “B”, fue utilizado “El cuestionario de Rendimiento Laboral Individual” el cual fue creado por Koopmans y colaboradores en el año 2013 en Holanda. Validado en Argentina por Gabini y Salessi en 2016 y traducido al español^15^. Posteriormente, validado en el Perú por los investigadores, obteniendo un índice de confiabilidad de Alfa de Cronbach de 0,690 y con un puntaje de V de Aiken de 0,810 de validez por juicio de expertos, por lo que el instrumento, muestra un índice de nivel de aceptable para su aplicación. El cuestionario consta de 16 ítems dividido en 3 dimensiones: Rendimiento en la tarea (7 ítems), Comportamientos contraproducentes (5 ítems) y Rendimiento en el contexto (4 ítems) con formato de respuesta tipo Likert de 5 puntos, significando 0= nunca, 1 = casi nunca, 2 = algunas veces, 3 = casi siempre y 4 = siempre.

### Procedimiento

Para la realización de la investigación el proyecto se presentó al comité de ética de la Facultad de Ciencias de la Salud de la Universidad Peruana Unión, brindándonos la autorización, contando también con la autorización de la institución educativa privada para realizar la ejecución del estudio. Para la aplicación del instrumento se utilizó la herramienta virtual Google Forms, donde los participantes respondieron las encuestas respectivas, en un tiempo aproximado de 30 minutos; con previa aceptación del consentimiento informado.

Los datos recolectados fueron procesados con el paquete estadístico R de acceso libre. Para el análisis univariado, se emplearon tablas de frecuencia simple; y para el bivariado, tablas de contingencia y la prueba Chi2, previa aplicación de la prueba de normalidad Kolmogorv-Smirnov para muestras superiores a 50, obteniendo un p-valor de 0,00, que, al ser menor a un nivel de significancia de 0,05, no sigue una distribución normal. Para el análisis multivariado, se utilizó la regresión logística binaria, considerando como variable dependiente al rendimiento laboral: alto (1), bajo (0).

## Resultados

De un total de 120 encuestados, más de la mitad fueron del sexo femenino, del grupo etario adulto, con un nivel de estudios de técnico y/o universitario, con un estado civil de casado y con hijos. Por otro lado, la mayoría procedían de la región Costa, trabajaban en el área de servicios generales, llevaban laborando en la institución entre 1 y 5 años, contaban con el antecedente de haber tenido COVID-19 y contaban con su esquema de vacunación completo contra el COVID-19 (Tabla 1).

Al análisis descriptivo de las variables, se halló que, de los 120 trabajadores el 52,50% de los encuestados tuvieron una buena percepción de los factores psicosociales, al igual que sus dimensiones: condición del lugar de trabajo, carga de trabajo, contenido y características de la tarea, papel laboral y desarrollo de la carrera, e Interacción social y aspectos organizacionales, en el 67,50%, 70,80 %, 80,00 %, 65,80% y 77,50%, respectivamente. Sin embargo, se obtuvo una mala percepción en las dimensiones exigencia laboral con el 63,30% y satisfacción con la remuneración del rendimiento con el 52,50%. Con relación a la variable rendimiento laboral, el 75,00% la calificó como alta, del mismo modo que en las dimensiones rendimiento de tarea con un 99,20% y rendimiento de contexto con un 72,50%. Por el contrario, la dimensión comportamientos contraproducentes obtuvo una baja puntuación en el 84,20% (Tabla 2).

**Tabla 1:** Análisis Descriptivo de las Variables de Estudio

Variables	n	%	n	%
	Malo		Buena	
Factores Psicosociales	57	47,50	63	52,50
Condición del lugar de trabajo	39	32,50	81	67,50
Carga de trabajo	35	29,20	85	70,80
Contenido y características de la tarea	24	20,00	96	80,00
Exigencias laborales	76	63,33	44	36,70
Papel laboral y desarrollo de la carrera	41	34,20	79	65,83
Interacción social y aspectos organizacionales	27	22,50	93	77,50
Satisfacción con la remuneración del rendimiento	63	52,50	57	47,50
		Alto		Bajo
Rendimiento laboral	90	75,00	30	25,00
Rendimiento en la tarea	119	99,20	1	0,80
Comportamientos contraproducentes	19	15,80	101	84,20
Rendimiento en el contexto	87	72,50	33	27,50

Al análisis bivariado, se halló que los factores sexo, antecedente de COVID-19, factores psicosociales y sus dimensiones tuvieron relación con el rendimiento laboral de los colaboradores de una institución educativa, con un p-valor menor a 0,05. Sin embargo, la edad, el grado de instrucción, el estado civil, el lugar de procedencia, el área de trabajo, el tiempo laborando en la institución y la vacunación contra el COVID-19 no tuvieron relación con el rendimiento laboral, con un p-valor mayor a 0,05 (Tabla 2).

Por último, al análisis multivariado, se encontró que sólo la dimensión exigencia laboral de los factores psicosociales estuvo asociado al rendimiento laboral (OR:11,03; IC 95%: 1,71-17,95). Por lo tanto, los colaboradores que indicaron una alta exigencia laboral tuvieron 11 veces mayor probabilidad de presentar un alto rendimiento en su trabajo (Tabla 2).

**Tabla 2:** Relación entre los factores psicosociales y rendimiento laboral en colaboradores de una institución educativa privada, Lima, 2021.

Variables	%n 120	Alto 90 Rendimiento laboral	Bajo 30 Rendimiento laboral	OR	(IC 95%)	Valor
Sexo						
Femenino	51,66(62)	57,77(52)	33,33(10)	1		
Masculino	48,33(58)	42,22(38)	66,66(20	0,82	(0,33-2,05)	0,67
Edad						
Joven	30,00 (36)	26,66 (24)	40,00 (12)	1		
Adulto	70,00 (84)	73,33 (66)	60,00 (18)	1,01	(0,28-3,59)	0,98
Grado de instrucción						
Secundaria completa	22,50 (27)	22,22 (20)	23,33 (7)	1		
Técnico/Universitario	77,50 (93)	77,78 (70)	76,67 (23)	0,61	(0,16-2,33)	0,47
Estado civil						
Soltero/Viudo/Divorciado	40,83 (49)	41,11 (37)	40,00 (12)	1		
Casado	59,17 (71)	58,89 (53)	60,00 (18)	2,10	(0,43-10,06)	0,35
Lugar de procedencia						
Costa	40,00 (48)	41,11 (37)	36,67 (11)	1		
Sierra	35,00 (42)	35,56 (32)	33,33 (10)	0,37	(0,98-1,39)	0,14
Selva	25,00 (30)	23,33 (21)	30,00 (9)	0,35	(0,09-1,32)	0,12
Area de trabajo						
Docente	31,67 (38)	34,44 (31)	23,33 (7)	1		
Administrativo	30,83 (37)	31,11 (28)	30,00 (9)	1,85	(0,60-5,67)	0,28
Servicios generales	37,50 (45)	34,44 (31)	46,67 (14)	0,80	(0,32-3,10)	0,99
Tiempo laborando en la institución						
Menos de 1 año	28,33 (34)	28,89 (26)	26,67 (8)	1		
De 1 a 5 años	52,50 (63)	48,89 (44)	63,33 (19)	3,83	(0,59-24,84)	0,16
Más de 5 años	19,17 (23)	22,22 (20)	10,00 (3)	0,39	(0,11-1,41)	0,15
¿Tiene hijos?						
Sí	58,33 (70)	57,78 (52)	60,00 (18)	1		
No	41,67 (50)	42,22 (38)	40,00 (12)	0,33	(0,07-1,52)	0,15
¿Usted tiene o ha tenido COVID-19?						
Sí	61,67 (74)	55,56 (50)	80,00 (24)	1		
No	38,33 (46)	44,44 (40)	20,00 (6)	0,89	(0,31-2,55)	0,84
¿Usted ha recibido la vacuna contra la COVID-19?						
Sí	97,50 (117)	97,78 (88)	96,67 (29)	1		
No	2,50 (3)	2,22 (2)	3,33 (1)	0,82	(0,02-31,09)	0,91
Factores psicosociales						
Alto	47,50 (57)	57,78 (52)	16,67 (5)	1		
Bajo	52,50 (63)	42,22 (38)	83,33 (25)	1,00	(0,96-1,05)	0,85
Condición del lugar de trabajo						
Alto	32,50 (39)	38,89 (35)	13,33 (4)	1		
Bajo	67,50 (81)	61,11 (55)	86,67 (26)	0,36	(0,09-1,42)	0,14
Carga de trabajo						
Alto	29,17 (35)	35,56 (32)	10,00 (3 )	1		
Bajo	70,83 (85)	64,44 (58)	90,00 (27)	1,45	(0,40-5,21)	0,56
Contenido y características de la tarea						
Alto	20,00 (24)	24,44 (22)	6,67 (2)	1		
Bajo	80,00 (96)	75,56 (68)	93,33 (28)	3,81	(0,60-24,18)	0,15
Exigencias laborales						
Alto	63,33 (76)	77,78 (70)	20,00 (6)	1		
Bajo	36,67 (44)	22,22 (20)	80,00 (24)	11,03	(1,71-17,95)	0,01
Papel laboral y desarrollo de la carrera						
Alto	34,17 (41)	40,00 (36)	16,67 (5)	1		
Bajo	65,83 (79)	60,00 (54)	83,33 (25)	0,31	(0,07-1,28)	0,10
Interacción social y aspectos organizacionales						
Alto	22,50 (27)	27,78 (25)	6,67 (2)	1		
Bajo	77,50 (93)	72,22 (65)	93,33 (28)	0,20	(0,03-1,12)	0,06
Satisfacción con la remuneración del rendimiento						
Alto	52,50 (63)	63,33 (57)	20,00 (6)	1		
Bajo	47,50 (57)	36,67 (33)	80,00 (24)	0,89	(0,22-3,47)	0,86

## Discusión

En la actualidad existen cambios que han predispuesto nuevos riesgos sobre la exposición a factores psicosociales negativos en el ambiente laboral que pueden dañar la salud y la calidad de vida de una persona. En la investigación presente se descubrió que el 63,30% de los trabajadores con “exigencias laborales” tuvieron factores psicosociales bajo o negativos, mientras que el 52,50% que tenía “satisfacción con la remuneración del rendimiento” presentaron factores psicosociales negativos (Tabla 2).

Resultados semejantes se encontraron en un estudio hecho por Pando en México^14^ del cual, un 58,70% tenían el factor psicosocial negativo con mayor predominio en las “exigencias laborales”. La literatura resalta que, al identificar las incidencias de los factores psicosociales frente al rendimiento laboral, se observa que estos están relacionados entre sí, influyendo significativamente en el desempeño de los trabajadores^18^. Por tanto, al identificar adecuadamente los factores psicosociales a tiempo en una institución laboral, donde aún no se han encontrado acontecimientos lamentables o consecuencias irremediables; es necesario presentar los riesgos laborales, su repercusión, y manifestar recomendaciones para promover adecuadamente la salud en el trabajo como método para enriquecer, aportar calidad y mejorar la salud pública de la población, y llevar a cabo acciones positivas de especialistas capacitados en materiales de prevención. Además, al tener conocimiento de lo son los factores de psicosociales de alto riesgo y de mayor incidencia en el centro de trabajo, contribuye a los niveles directos del crecimiento de una entidad.

De igual forma, a los expertos en el área de seguridad y salud ocupacional en el trabajo, atribuye a planear y plantear estrategias o programas de prevención con finalidad de deshacer los factores psicosociales negativos que se presentan en el área laboral^19^.

De acuerdo, a lo anterior señalado en la investigación se descubrió que el 80% de los trabajadores que tienen o han tenido COVID-19 presentan bajo rendimiento laboral, mientras que un 55,60% si evidenciaba alto rendimiento laboral. Asimismo, el 83,33% de empleados que tenían factores psicosociales presentaba bajo rendimiento laboral. Resultados similares se reportaron en Barcelona - España donde 65,1% presentaron bajo rendimiento laboral frente al contexto de la COVID-19^19^. En torno a esto, según Benítez^20^ alega que la crisis sanitaria por COVID - 19, ha generado grandes cambios en diferentes aspectos, de los cuales la adaptación a nuevas formas de trabajo, que suele incitar aumento en la carga laboral, familiar y mental.

También, menciona que este factor psicosocial mantiene relación con el desempeño del trabajador, debido a que este escenario de la pandemia por la COVID-19, por intento de prevenir la disminución excesiva del rendimiento laboral, los trabajadores tengan que realizar sus labores, con exposición al virus, el miedo a la exposición, el estrés, ansiedad del confinamiento y la cuarentena; provocan que existe menos productividad laboral, por consiguiente, menos rendimiento laboral. Asimismo, los factores psicosociales negativos son una problemática que puede limitar el rendimiento laboral de los individuos, generando menos eficacia, productividad y abandono o deseos en el trabajo.

Otro resultado, con significancia que muesWtra el estudio, fue que el 77,80% de trabajadores tienen altas exigencias laborales de los factores psicosociales se asocian a un alto rendimiento laboral. El rendimiento laboral indica la productividad del personal en su puesto de trabajo, para lo cual, la organización busca buenos resultados a través de ella. La literatura^21^ menciona que existen diferentes tipos de factores que predominan en el rendimiento laboral, una de ellas es la exigencia laboral o el deber y la importancia que desempeña un empleado en su trabajo es primordial para realizar correctamente sus funciones, ejerciendo los conocimientos, habilidades y la experiencia suficiente para garantizar su permanencia en una institución. Asimismo, el implementar objetivos desarrollados en un lapso de tiempo provee al empleado cumplir con el reto.

### Aspectos éticos

El presente estudio se ejecutó luego de obtener la carta de autorización concerniente al comité de ética de la Facultad de Ciencias de la Salud de la Universidad Peruana Unión número 2021-CE-FCS - UPeU-00268 y de la institución privada a intervenir. Con la finalidad de cumplir con el principio privacidad humana y respeto a la dignidad, los investigadores brindamos la información importante para la colaboración voluntaria de cada participante a estudiar, por medio de una estricta privacidad, anonimato y confidencialidad para el empleo de los datos obtenidos, con la finalidad de efectuar los objetivos planteados en el estudio. Asimismo, con el propósito de mantener los principios y un estricto marco ético, por medio de la cual, se elaboró un consentimiento informado para proteger la integridad y agilizar la libre respuesta del participante. Por consiguiente, queda garantizado el uso confidencial de la información obtenida en esta investigación.

### Recomendaciones

Si bien el 52,5% de los encuestados tuvieron una buena percepción de los factores psicosociales, se sugiere que la institución en estudio, mejore las condiciones y factores psicosociales presentes, principalmente, las dimensiones exigencia laboral y satisfacción con la remuneración, elaborando estrategias (programas, talleres, capacitaciones, etc.), que implique el manejo para el mejoramiento en el rendimiento laboral. Realizar estudios que profundicen el análisis de los factores psicosociales en el trabajo que afectan significativamente la salud de los trabajadores.

## Conclusiones

El estudio concluye, en que existe una relación directa y relevante entre las variables de la investigación Factores psicosociales y rendimiento laboral y; asimismo, más de la mitad de los trabajadores de la institución privada en estudio, presentaron factores psicosociales en un nivel alto y también poseen alto rendimiento laboral. Sin embargo, debe considerarse que el estudio fue predominantemente en una muestra femenina y en trabajadores de una institución privada.

Identificar, analizar y evaluar las causas de los factores psicosociales que afectan el rendimiento laboral como parte de la política constitucional de la organización.

Continuar con la línea de investigación para mejora de la legislación laboral en beneficio de los trabajadores.
